# Activated CD4 + T lymphocyte is a potential biomarker for acute graft-vs.-host disease after hematopoietic stem cell transplantation in children with transfusion-dependent β-thalassemia

**DOI:** 10.3389/fped.2022.985306

**Published:** 2022-09-29

**Authors:** Ken Huang, Jianming Luo

**Affiliations:** Department of Pediatrics, The First Affiliated Hospital of Guangxi Medical University, Nanning, China

**Keywords:** lymphocyte subsets, acute graft-vs.-host disease, HSCT, thalassemia, biomarker

## Abstract

**Background:**

Acute graft-vs.-host disease (aGVHD) is still one of the most common and life-threatening complications of allogeneic hematopoietic stem cell transplantation (HSCT). Whether or not the level of activated T lymphocytes rises before the onset of aGVHD is unknown. We explored the possibility of T lymphocytes as biomarkers for early prediction of aGVHD in children with transfusion-dependent β-thalassemia (TDTβ).

**Methods:**

We retrospectively analyzed the characteristics of T lymphocyte subsets before and 14 days after HSCT in children with TDTβ who developed aGVHD. Data from 95 children (Age ≤ 14 years) who underwent allogeneic HSCT from January 2020 to December 2021 were collected. Patients were divided into non-aGVHD group (*n* = 55) and aGVHD group (*n* = 40), and aGVHD group was divided into two subgroups: grade I aGVHD (*n* = 16) and grade II-IV aGVHD (*n* = 24). Receiver operating characteristic curve (ROC) analysis was performed to predict aGVHD.

**Results:**

Before preconditioning in non-aGVHD and aGVHD groups, there was no significant difference in all lymphocyte subsets and ratio of CD4 + /CD8 + T cells. On day 14 post-transplantation in non-aGVHD and aGVHD groups, the absolute concentrations per μl blood of T cells, CD4 + T cells, CD8 + T cells, activated CD4 + T cell and NK cells, were 69.73 (14.70, 137.77) and 140.36 (65.06, 293.42), 10.00 (2.35, 23.59) and 35.91 (12.41, 68.71), 37.25 (5.82, 84.36) and 89.99 (35.83, 180.81), 0.52 (0.17, 2.20) and 4.08 (0.91, 11.12), 43.86 (15.00, 91.31) and 26.35 (15.19, 49.39), respectively. On day + 14 (14 days post-transplantation), the differences in all cell subsets and the ratio of CD4 + /CD8 + T cells were not statistically significant between grade I aGVHD and grade II-IV aGVHD subgroups. The absolute concentrations of CD8 + T cells in grade I aGVHD were significantly higher than in grade II-IV aGVHD [128.21 (61.11, 258.91) vs. 60.81 (21.59, 176.38), *P* = 0.057]. AUC of NK cells, CD8 + T cells, T cells, CD4 + T cells, and CD4 + CD25 + T cells were 0.6275, 0.6839, 0.7068, 0.7241, and 0.7589, and cut-off values were 73.75 (97.50, 34.55), 146.90 (37.50, 94.55), 187.30 (45.00, 90.91), 18.95 (70.00, 72.73), and 3.24 (52.50, 87.27), respectively. The AUC of the combined CD4 + CD25 + T cells and CD8 + T cells, CD4 + CD25 + T cells and T cells, CD4 + CD25 + T cells and CD4 + T cells, CD4 + CD25 + T cells and NK cells, respectively, were 0.7500, 0.7598, 0.7750, and 0.8050.

**Conclusion:**

Our findings demonstrate that level of activated CD4 + T cells on day + 14 (post-HSCT) is a valuable biomarker for predicting aGVHD in children with TDTβ and CD8 + T cells could likely be a biomarker for severe aGVHD.

## Introduction

Thalassemia is an autosomal recessive blood disease characterized by anemia that develops because of the damaged synthesis of one or more of the hemoglobin chains (1). β-thalassemia (TDTβ) is caused by mutations in the gene encoding β-chains of the hemoglobin (Hb) and is characterized by the reduced or absent synthesis of β-globin, ineffective erythropoiesis, and hemolysis of mature red blood cells (RBCs) caused by excess α-chains (2). Thalassemia is divided into blood transfusion-dependent thalassemia (TDT) and non-transfusion-dependent thalassemia (NTDT). Each year, there are more than 40,000 babies born with TDTβ worldwide, 26,000 of which have TDT, most babies with TDTβ are born in resource-constrained countries (2). It is a serious threat to human health and a public health problem around the world.

Transfusion-dependent TDTβ can be treated through regular transfusion of blood, iron chelation management, hematopoietic stem cell transplantation (HSCT), stimulation of fetal hemoglobin production, and gene therapy (3). Despite the potential of gene therapy to completely cure TDTβ, cost management, and long-term safety limit its clinical application (4). HSCT is currently the only method that can be promoted to cure thalassemia. Despite significant advances in prophylaxis and therapy, acute graft vs. host disease (aGVHD) remains one of the most common and life-threatening complications of allogeneic HSCT (5), resulting in considerable morbidity and mortality (6). The cumulative incidence of grade II-IV aGVHD in children who have received allogeneic HSCT ranges from 28 to 56% (7–9). Early intensifying immunosuppression, however, puts patients at high risk of infectious complications, whereas late treatment often fails to prevent disease exacerbation (10). There is consensus that accurate risk prediction and early diagnosis of aGVHD could significantly improve patient outcomes. Current approaches to aGVHD risk assessment are poorly standardized across countries and medical centers (11). Most current recommendations depend on clinical factors that are insufficient or difficult to use for precise risk stratification, such as the applied conditioning regimen, donor-recipient relationship, and HLA match status. The risk assessment of aGVHD for a single disease is rarely reported. Until now, no predictive models have been established for aGVHD following HSCT in children with transfusion-dependent thalassemia (TDT). Accurate risk prediction is an unmet clinical need.

As far as we know, aGVHD occurs when cells from the graft recognize minor histocompatibility antigens expressed on non-hematopoietic cells, and cause damage in tissues—typically gut, liver, and skin (12). The pathophysiology of GvHD is currently felt to occur through several phases (13–15). In the first phase, damage by the chemotherapy or radiotherapy used in the transplant preparatory regimen causes host tissues to secrete inflammatory cytokines (16). This results in the activation of alloreactive donor T-cells that recognize HLA and minor histocompatibility antigen disparities on host cells (16). Subsequently, the donor T cells and other immune effectors elaborate a variety of inflammatory cytokines including TNF-α, IFN-γ, IL-13, IL-5, and others resulting in the widespread tissue damage observed clinically (16). B cells and natural killer cells may also play important roles in GVHD through additional mechanisms (17–19). GVHD occurs when cells from the graft recognize minor histocompatibility antigens expressed on non-hematopoietic cells, and cause damage in tissues—typically gut, liver, and skin. T lymphocytes and cytokines play pivotal roles in the occurrence and development of aGVHD. Based on this knowledge, over the last few decades, considerable research efforts have been devoted to identifying and validating novel and reliable molecular biomarkers for aGVHD diagnosis, prognosis, risk assessment, and prediction of therapy response (20). Many studies have found that serum cytokine CD25 increased significantly before the occurrence of aGVHD, which can be an early predictor of aGVHD (21–27). However, whether CD25 + T cells have similar predictive value is still unclear. Additionally, there are few reports on the changes of T lymphocyte subsets before the onset of acute graft- vs.-host disease after HSCT in children with TDT. Here, we retrospectively analyzed the characteristics of T lymphocyte subsets (including CD4 + CD25 + T lymphocytes) before preconditioning and 14 days after transplantation in TDT children who developed aGVHD, and explore the possibility of using T-lymphocyte subsets as biomarkers for early prediction of aGVHD, in order to provide a reference for the early warning of aGVHD in children with thalassemia.

## Patients and methods

### Patients

Data of children (Age ≤ 14 years) with thalassemia who underwent allogeneic HSCT in the First Affiliated Hospital of Guangxi Medical University from January 1, 2020 to December 31, 2021, were collected. Patients were divided into non-aGVHD group and aGVHD group according to whether aGVHD occurred. The aGVHD group was divided into two subgroups: grade I aGVHD and grade II-IV aGVHD. Inclusion criteria: ➀ Age ≤ 14 years; ➁ diagnosed as transfusion-dependent TDTβ; ➂ all cases had no underlying diseases, including leukemia, lymphoma, aplastic anemia, Langerhans cell histiocytosis, and other hematological diseases; ➃ No monoclonal antibody against CD25 was administered before day + 14 post-transplantation. This project was approved by the Medical Ethics Committee of the First Affiliated Hospital of Guangxi Medical University [NO.2022-KY-E-(214)].

### Diagnosis and grading of acute graft-vs.-host disease

Diagnosis and grading of aGVHD were based on the clinical and pathological features of the patient, in accordance with the International Federation of Acute Graft- vs.-Host Disease (the Mount Sinai Acute GVHD International Consortium, MAGIC) standards (28).

### Conditioning regimen

All patients in this study were treated with a conditioning regimen of busulfan (Bu) + cyclophosphamide (Cy) + fludarabine (Flu) + anti-human thymocyte globulin (ATG). The details are as follows: (1) Bu 1 mg/kg 4 times daily on day –9 and –6; (2) Cy 50 mg/kg once daily on day –5 to –2; (3) Flu 50 mg/m^2^ once daily on day –12 to –9; (4) ATG 10 mg/kg once daily on day –5 to –2. All patients were given hydroxyurea 20 mg/kg once daily orally for 2–3 months prior to transplantation.

### Graft-vs.-host disease prophylaxis

All patients whose donor were related match received a standard immunosuppressive GVHD prophylaxis regimen consisting of Cyclosporine (if HLA matched sibling donor transplantation) or tacrolimus (if related mismatched or unrelated donor transplantation), mycophenolate mofetil (MMF), and short-term methotrexate. Cyclosporine (intravenous, IV) was initiated at day –1 at a dose of 3 mg/kg/day, blood cyclosporine trough level was done twice weekly to maintain the level between 150 and 250 ng/ml. When the patient began to tolerate oral feeding, cyclosporine was shifted to oral route. Tacrolimus (TAC) (IV) was used 1 day prior to transplantation. The initial dosage of TAC was 0.015 mg/kg, twice daily, with intravenous infusion administered over a period of 2 h. Subsequent dosages were adjusted based on the patients’ condition and the plasma concentrations achieved. For patients tolerating oral administration, intravenous TAC was switched to oral TAC. MMF (250 mg/day) given 1 day before transplantation to 30 days after transplantation. Methotrexate (IV) was given at day + 1 with a dose of 15 mg/kg, then 10 mg/kg was given at days + 3, + 6 and + 11. Rescue folic acid (IV) at a dose of 15 mg/kg was given 24 h following each dose of methotrexate.

### Flow cytometry

Lymphocyte subsets including T cells (CD3 +), CD4 + T cells (CD3 + CD4 + CD8-), CD8 + T cells (CD3 + CD4-CD8 +), activated CD4 + T cells (CD45 + CD4 + CD25 +), B cells (CD3-CD19 +), NK cells (CD3-CD16 + /CD56 +), double negative T cells (DN, CD3 + CD4-CD8-), and double positive T cells (DP, CD3 + CD4 + CD8 +) were detected before and 14 days after allogeneic HSCT. BD FACS Canto TM II flow cytometry, BD Multitest TM IMK Kit, FITC Mouse Anti-Human CD4, PerCP Mouse Anti-Human CD45, and PE Mouse Anti-Human CD25 were used. 5 ml peripheral venous blood was collected aseptically by venipuncture, using EDTA blood collection tubes. Three 12 × 75 mm tubes were labeled with letters A, B, and C for each sample. 20 μL of BD Multitest CD3-FITC/CD8-PE/CD45-PerCP/CD4-APC reagent was added into the bottom of tube labeled A and 20 μL of BD Multitest CD3-FITC/CD16 + CD56-PE/CD45-PerCP/CD19-APC reagent was added into the bottom of tube labeled B. Then 20 μL of each reagent of CD4-FITC, CD45-PerCP, and CD25-PE was added into the bottom of tube labeled C. A total of 50 μL of well-mixed, anticoagulated whole blood was added into the bottom of each tube. The tubes were capped and vortexed gently to mix. The tubes were incubated for 15 min in the dark at room temperature (20–25°C). A total 450 μL of 1X BD Multitest IMK kit lysing solution was added to each tube. The tubes were capped and vortexed gently to mix. The tubes were incubated for 15 min in the dark at room temperature (20–25°C). Samples were analyzed within an hour after preparation. According to the double-parameter point plots of forward and side scattering light, “gate” was set on leukocyte common antigen (CD45) positive cells, 10,000 lymphocytes were counted, and the surface markers of lymphocytes were analyzed with double-parameter. The percentages of T cells, CD4 + T cells, CD8 + T cells, CD4 + CD25 + T cells, B cells, NK cells, DN, DP and ratio of CD4 + /CD8 + T cells were calculated. And then absolute concentrations per μl blood were calculated based on the percentages of T cell subsets and the lymphocyte concentrations in the corresponding blood routine tests. All data were analyzed by flow cytometry and BD FACSCanto clinical software.

### Statistical analysis

All statistical analyses were conducted using SPSS software (version 26.0) and Graph Pad Prism (version 9.0). Normality tests were performed on all measurement data. Measurement data that conformed to a normal distribution are presented as mean ± SD, and an independent sample *T*-test was used to calculate differences between groups of patients. Measurement data that did not conform to the normal distribution are represented as median (interquartile) [range], and the Mann-Whitney *U*-test was used to calculate differences between groups of patients. Categorical data are presented as n (percentage) and analyzed using chi-square and Fisher’s exact test. Pearson correlation (r) was used to assess the association between T lymphocyte pairs with statistical significance in univariate analysis. To determine a predictive significance receiver operating-characteristic curve (ROC) analysis was performed. A two-sided *P*<0.05 was considered statistically significant.

## Results

### Characteristics of patients and transplantations

Characteristics of patients and transplantations of 95 patients analyzed are listed in Tables 1, 2. A total of 95 children under the age of 14 with TDTβ who underwent allogeneic HSCT in the First Affiliated Hospital of Guangxi Medical University from January 1, 2020 to December 31, 2021, were included. There were no primary or secondary graft failures and no recurrances of TDTβ in all cases. Among them, 17 cases were combined with mild to moderate α-thalassemia. There were 55 cases in the control group and 40 cases in the experimental group (16 cases in subgroup 1 and 24 cases in subgroup 2). The median recipient age was 8.0 years and 65% were male. All unrelated donors were compatible with recipients (HLA10/10). 25 (26.3%) cases were mismatched related donors, and 16 (16.8%) cases were haploid among them. The percentages of different types of stem cell sources were 5.2% (Bone marrow), 40.0% (Peripheral blood), 7.4% (Bone marrow + Cord blood), and 47.4% (Bone marrow + Peripheral blood), respectively. The median input of total monocytes and CD34 + stem cells were 13.05 (9.15, 15.75) × 10^8^/kg and 9.19 (6.45, 12.32) × 10^6^/kg, respectively. The median engraftment time of neutrophil was 12.0 (11.0, 13.0) [8.0, 23.0] days. Platelet transfusion was ineffective in 2 patients with positive platelet antibodies, and platelet level was not lower than 20 × 10^9^/L in 4 patients after transplantation. In the other 89 cases, the median time of platelet engraftment was 13 (11. 15) [8.0, 28.0] days. The median time from stem cell transplantation to aGVHD onset was 28.0 (17.0, 36.8) [11.0, 83.0] days. The number of bacterial, viral, fungal, bacterial + viral and bacterial + fungal infections in aGVHD group were 10 (25.0%), 8 (20.0%), 1 (2.5%), 3 (7.5%), and 1 (2.5%) within the peri-transplantation period and 30 days post-transplantation (d-12 to day + 30), respectively. The number of bacterial, viral, fungal, bacterial + viral and bacterial + fungal infections in non-aGVHD group were 15 (27.3%), 4 (7.3%), 2 (3.6%), 1 (1.8%), 1 (1.8%), and 32 (58.2%), respectively. There was no significant difference in the composition ratio of infections between the aGVHD and non-aGVHD groups (*P* = 0.302). Of the 40 cases of acute graft- vs.-host disease, 16 (40.0%) were grade I, 9 (22.5%) were grade II, 8 (20.0%) were grade III, 7 (17.5%) were grade IV, and 24 (60.0%) were grade II-IV in total. The organs involved were skin 18 (45.0%), gut 9 (22.5%), skin + liver 1 (2.5%), skin + gut 12 (30.0%), and skin + gastrointestinal tract + liver 0 (0%). All patients with intestinal involvement underwent colonoscopic biopsies to determine intestinal aGVHD.

**TABLE 1:** Characteristics of patients and transplantations.

Variables	Total (*n* = 95)	aGVHD (*n* = 40)	Non- aGVHD (*n* = 55)	*P*
Age at HSCT, median (IQR)	8.0 (5.3, 11.2)	9.3 (6.6, 11.9)	7.3 (4.6, 9.6)	0.006
[Range], *y*	[2.4, 14.0]	[3.7, 14.0]	[2.4, 13.0]	
**Sex, *n* (%)**				
Male	65 (68.4)	29 (72.5)	36 (65.5)	0.466
Female	30 (31.6)	11 (27.5)	19 (34.5)	
**Genotype of thalassemia, *n* (%)**				
β0/β0	60 (63.2)	19 (47.5)	41 (74.5)	0.004
β0/non-β0	30 (31.6)	20 (50.0)	10 (18.2)	
non-β0/non-β0	5 (5.2)	1 (2.5)	4 (7.3)	
Simultaneous α globin mutation	17 (17.9)	4 (10.0)	13 (23.6)	0.087
**Donor–recipient gender match, *n* (%)**				
Female to female	11 (11.6)	3 (7.5)	8 (14.6)	0.758
Female to male	23 (24.2)	10 (25.0)	13 (23.6)	
Male to female	19 (20.0)	8 (20.0)	11 (20.0)	
Male to male	42 (44.2)	19 (47.5)	23 (41.8)	
**Donor type, *n* (%)**				
Matched related	32 (33.7)	5 (12.5)	27 (49.1)	0.001
Matched unrelated	38 (40.0)	22 (55.0)	16 (29.1)	
Mismatched related	25 (26.3)	13 (32.5)	12 (21.8)	
**ABO mismatch, *n* (%)**				
None	44 (46.3)	16 (40.0)	28 (50.9)	0.729
Minor	18 (18.9)	8 (20.0)	10 (18.2)	
Major	26 (27.4)	13 (32.5)	13 (23.6)	
Bidirectional	7 (7.4)	3 (7.5)	4 (7.3)	
**Stem cell source, *n* (%)**				
BM	5 (5.2)	1 (2.5)	4 (7.3)	0.016
PB	38 (40.0)	22 (55.0)	16 (29.1)	
BM + CB	7 (7.4)	0 (0.0)	7 (12.7)	
BM + PB	45 (47.4)	17 (42.5)	28 (50.9)	
**Graft**				
MNC median (IQR)	13.05 (9.15, 15.75)	13.11 (9.95, 15.90)	12.45 (8.75, 15.73)	0.670
[range] × 108/kg	[1.06, 29.81]	[5.19, 28.65]	[1.06, 29.81]	
CD34 + cells, median, (IQR)	9.19 (6.45, 12.32)	7.88 (6.77, 11.07)	10.56 (5.60, 14.07)	0.246
[range] × 106/kg	[1.44, 25.51]	[4.63, 24.36]	[1.44, 25.51]	
**Engraftment**				
Neutrophil, median (IQR)	12.0 (11.0, 13.0)	11.0 (11.0, 12.0)	12.0 (10.0, 14.0)	0.295
[range], days	[8.0, 23.0]	[8.0, 15.0]	[8.0, 23.0]	
Platelet, median (IQR)	13.0 (11.0, 15.0) *	13.0 (11.0, 15.0) ^▲^	12.5 (11.0, 15.0) ^●^	0.764
[Range], days	[8.0, 28.0]	[8.0, 28.0]	[9.0, 20.0]	
**Infections peri- and post-HSCT^◆^, *n* (%)**				
Bacterial	25 (26.3)	10 (25.0)	15 (27.3)	0.302
Viral	12 (12.6)	8 (20.0)	4 (7.3)	
Fungal	3 (3.2)	1 (2.5)	2 (3.6)	
Bacterial + viral	4 (4.2)	3 (7.5)	1 (1.8)	
Bacterial + fungal	2 (2.1)	1 (2.5)	1 (1.8)	
None	49 (51.6)	17 (42.5)	32 (58.2)	

HSCT, Hematopoietic stem cell transplantation; IQR, Interquartile range; BM, Bone marrow PB, Peripheral blood; CB, Cord blood; MNC, Mononuclear cells; *Platelet transfusion was ineffective in 2 patients with positive platelet antibody, and platelet level was not lower than 20 × 109/L in 4 patients after transplantation, *n* = 89; ^▲^*n* = 50; ^●^*n* = 39; ^◆^Duration from day-12 before transplantation to day + 30 post-transplantation.

**TABLE 2:** Features of acute graft- vs.-host disease (aGVHD).

Variables	Value
Total, *n*	40
**Grade, *n* (%)**	
Grade I	16 (40.0)
Grade II–IV	24 (60.0)
Grade II	9 (22.5)
Grade III	8 (20.0)
Grade IV	7 (17.5)
Affected organs by aGVHD, *n* (%)*	
Skin	18 (45.0)
Gut	9 (22.5)
Skin + Liver	1 (2.5)
Skin + Gut	12 (30.0)
Time from stem cell transplantation to aGVHD onset median (IQR)[range], Days	28.0 (17.0, 36.8) [11.0, 83.0]

IQR, Interquartile range. *No case of skin + gut + liver.

### Analysis results of lymphocyte subsets before transplantation preconditioning and on day + 14 post-transplantation

**FIGURE 1:**
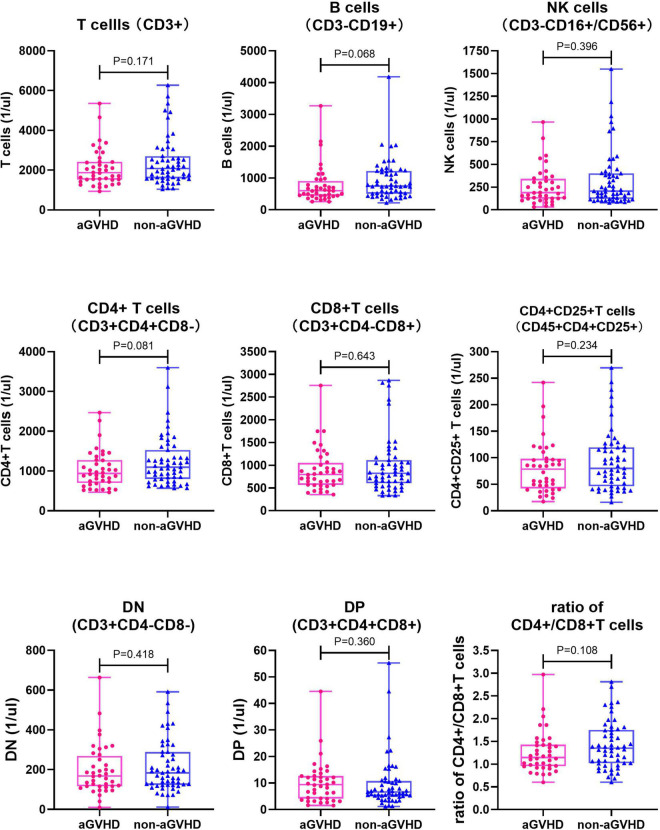
Comparison of each of lymphocyte subsets and ratio of CD4 + /CD8 + T cells between non-aGVHD and aGVHD groups before preconditioning (*n* = 95). NK cells, Natural killer cells; DN, Double negative T cells; DP, Double positive T cells.

**FIGURE 2:**
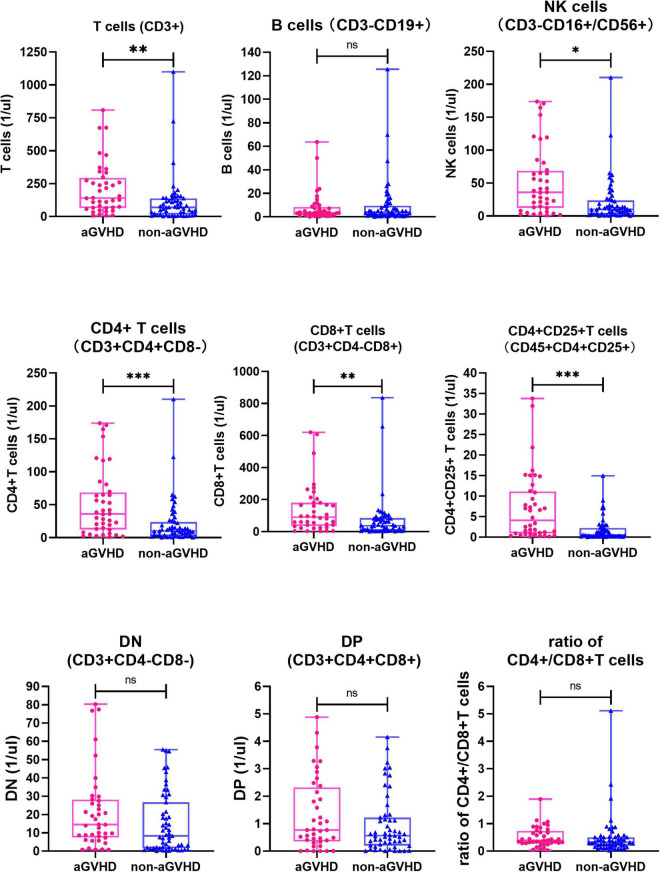
Comparison of each of lymphocyte subsets and ratio of CD4 + /CD8 + T cells between non-aGVHD and aGVHD groups on day + 14 post-transplantation (*n* = 95). NK cells, Natural killer cells; DN, Double negative T cells; DP, Double positive T cells. *^ns^P* ≥ 0.05; *P<0.05; ^**^P<0.01; ^***^P<0.001.

**FIGURE 3:**
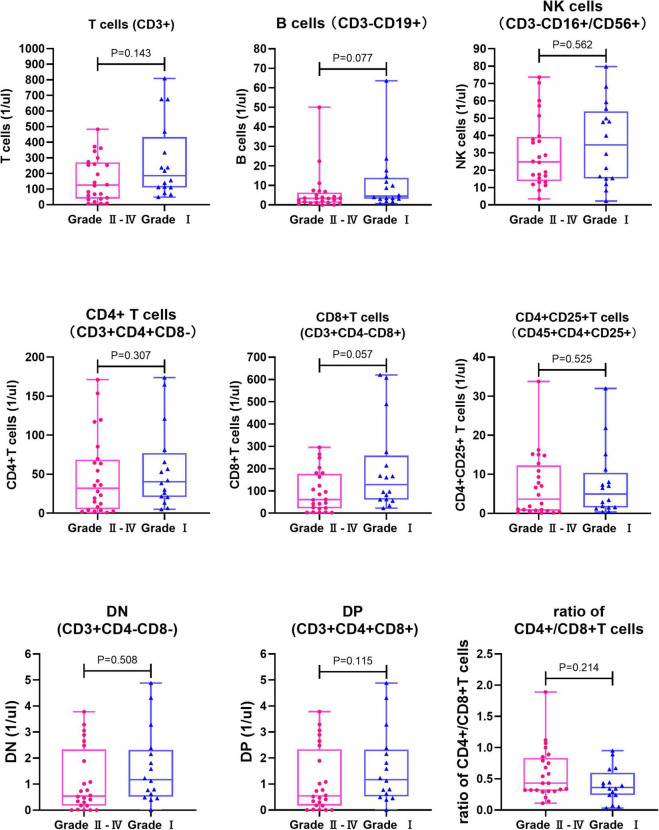
Comparison of lymphocyte subsets and ratio of CD4 + /CD8 + T cells between Grade I and Grade II-IV aGVHD subgroups on day + 14 post transplantation (*n* = 40). NK cells, Natural killer cells; DN, Double negative T cells; DP, Double positive T cells.

A total of 190 samples from 95 children before transplantation preconditioning and 14 days after transplantation were detected. There was no significant difference in the level of all lymphocyte subsets and the ratio of CD4 + /CD8 + T cells between the aGVHD group and the non-aGVHD group before transplantation preconditioning (Table 3 and Figure 1). On day + 14 post-transplantation in the non-aGVHD group and the aGVHD group, the absolute concentrations per μl blood of T cells, CD4 + T cells, CD8 + T cells, activated CD4 + T cell and NK cells, were 69.73 (14.70, 137.77) and 140.36 (65.06, 293.42), 10.00 (2.35, 23.59) and 35.91 (12.41, 68.71), 37.25 (5.82, 84.36) and 89.99 (35.83, 180.81), 0.52 (0.17, 2.20) and 4.08 (0.91, 11.12), 43.86 (15.00, 91.31) and 26.35 (15.19, 49.39), respectively. For all cell subsets, the differences between the aGVHD group and the non-aGVHD group were all statistically significant (*P* < 0.05). The levels of all cell subsets except for NK cells were higher in the aGVHD group than in the non-aGVHD group (Table 4 and Figure 2). In the non-aGVHD group and the aGVHD group, the absolute concentrations per μl blood of B cells, DN, DP, and ratio of CD4 + /CD8 + T cells were 3.86 (1.35, 9.44) and 3.45 (1.68, 8.33), 8.25 (1.59, 26.69) and 14.54 (7.49, 28.07), 0.56 (0.20, 1.22) and 0.77 (0.35, 2.32), 0.32 (0.20, 0.50) and 0.39 (0.29, 0.73), respectively. The differences in B cells, DN, DP and ratio of CD4 + /CD8 + T cells between the non-aGVHD group and the aGVHD group were not statistically significant (*P* ≥ 0.05). In the aGVHD group, on day + 14 post-transplantation, the level of CD8 + T cells in the grade I subgroup was significantly higher than that in grade II-IV [128.21 (61.11, 258.91) vs. 60.81 (21.59, 176.38)], but the difference was not statistically significant at the level of *P* < 0.05 (*P* = 0.057). The differences between T cells, CD4 + T cells, activated CD4 + T cells, B cells, NK cells, DN, DP, and the ratio of CD4 + /CD8 + T cells between grade I and grade II-IV subgroups were not statistically significant (Table 5 and Figure 3).

**TABLE 3:** Results of lymphocyte subsets between non-aGVHD and aGVHD groups before preconditioning of transplantation (*n* = 95).

Variables	Non-aGVHD	aGVHD	*P*
	(*n* = 55)	(*n* = 40)	
T cells (1/μl)	2084.12 (1592.79, 2705.48)	1871.30 (1513.50, 2418.39)	0.171
B cells (1/μl)	742.34 (506.02, 1222.35)	604.12 (439.40, 906.63)	0.068
NK cells (1/μl)	204.23 (120.24, 400.52)	189.38 (123.96, 340.46)	0.396
CD4 + T cells (1/μl)	1093.61 (798.69, 1528.88)	938.38 (698.42, 1275.79)	0.081
CD8 + T cells (1/μl)	820.66 (601.60, 1111.30)	798.65 (568.61, 1052.38)	0.643
CD4 + CD25 + T cells (1/μl)	79.99 (46.02, 119.70)	78.48 (42.24, 98.14)	0.234
DN (1/μl)	184.00 (122.77, 288.79)	167.94 (116.57, 269.03)	0.418
DP (1/μl)	6.63 (5.04, 10.79)	9.35 (4.10, 12.66)	0.360
Ratio of CD4 + /CD8 + T cells	1.35 (1.02, 1.75)	1.15 (0.95, 1.43)	0.108

NK cells, Natural killer cells; DN, Double negative T cells; DP, Double positive T cells.

**TABLE 4:** Results of lymphocyte subsets between non-aGVHD and aGVHD groups on day + 14 post transplantation (*n* = 95).

Variables	Non-aGVHD	aGVHD	*P*
	(*n* = 55)	(*n* = 40)	
T cells (1/μl)	69.73 (14.70, 137.77)	140.36 (65.06, 293.42)	0.001
B cells (1/μl)	3.86 (1.35, 9.44)	3.45 (1.68, 8.33)	0.845
NK cells (1/μl)	43.86 (15.00, 91.31)	26.35 (15.19, 49.39)	0.035
CD4 + T cells (1/μl)	10.00 (2.35, 23.59)	35.91 (12.41, 68.71)	0.000
CD8 + T cells (1/μl)	37.25 (5.82, 84.36)	89.99 (35.83, 180.81)	0.002
CD4 + CD25 + T cells (1/μl)	0.52 (0.17, 2.20)	4.08 (0.91, 11.12)	0.000
DN (1/μl)	8.25 (1.59, 26.69)	14.54 (7.49, 28.07)	0.054
DP (1/μl)	0.56 (0.20, 1.22)	0.77 (0.35, 2.32)	0.213
Ratio of CD4 + /CD8 + T cells	0.32 (0.20, 0.50)	0.39 (0.29, 0.73)	0.105

NK cells, Natural killer cells; DN, Double negative T cells; DP, Double positive T cells.

**TABLE 5:** Results of lymphocyte subsets between Grade I and Grade II-IV aGVHD subgroups on day + 14 post transplantation (*n* = 40).

Variables	Grade I	Grade II–IV	*P*
	(*n* = 16)	(*n* = 24)	
T cells (1/μl)	185.92 (110.50, 433.98)	125.02 (38.83, 270.55)	0.143
B cells (1/μl)	4.47 (3.08, 13.90)	3.33 (1.11, 6.39)	0.077
NK cells (1/μl)	34.61 (15.19, 53.98)	24.78 (13.80, 39.22)	0.562
CD4 + T cells (1/μl)	40.21 (20.76, 77.10)	31.87 (5.12, 68.53)	0.307
CD8 + T cells (1/μl)	128.21 (61.11, 258.91)	60.81 (21.59, 176.38)	0.057
CD4 + CD25 + T cells (1/μl)	4.94 (1.47, 10.40)	3.63 (0.65, 12.28)	0.525
DN (1/μl)	18.73 (6.61, 33.76)	13.34 (7.63, 26.90)	0.508
DP (1/μl)	1.17 (0.52, 2.32)	0.54 (0.17, 2.33)	0.115
Ratio of CD4 + /CD8 + T cells	0.36 (0.24, 0.60)	0.43 (0.31, 0.84)	0.214

NK cells, Natural killer cells; DN, Double negative T cells; DP, Double positive T cells.

### Correlation between lymphocyte subsets on day + 14 post transplantation

Pearson correlation test was performed for T cells, NK cells, CD4 + T cells, CD8 + T cells, and activated CD4 + T cells on day + 14 after transplantation (Table 6). There were strong correlations between T cells and CD8 + T cells, T cells and CD4 + T cells, CD4 + T cells and CD8 + T cells (r ≥ 0.7), moderate correlations between CD4 + T cells and CD4 + CD25 + T cells, T cells and CD4 + CD25 + T cells (0.50 ≤ *r* < 0.70), and low correlations between T cells and NK cells, CD8 + T cells and NK cells, CD8 + T cells and CD4 + CD25 + T cells (0.30 ≤ *r* < 0.50. All the correlations were positive. There were no correlations between CD4 + T cells and NK cells, CD4 + CD25 + T cells, and NK cells. All the above correlations were positive.

**TABLE 6:** The correlations among five subsets (T cells, CD4 + CD25 + T cells, CD4 + T cells, CD8 + T cells, and NK cells) on day + 14 post-transplantation.

	T cells	NK cells	CD4 + T cells	CD8 + T cells	CD4 + CD25 + T cells
T cells	1				
NK cells	0.304**	1			
CD4 + T cells	0.843**	0.16	1		
CD8 + T cells	0.978**	0.343**	0.726**	1	
CD4 + CD25 + T cells	0.513**	–0.054	0.605**	0.438**	1

NK cells, Natural killer cells. **At level 0.01 (double tail), the correlation was significant.

### Analyses of receiver operating-characteristic curve curves of lymphocyte subsets on day + 14 post transplantation predict acute graft-vs.-host disease

**FIGURE 4:**
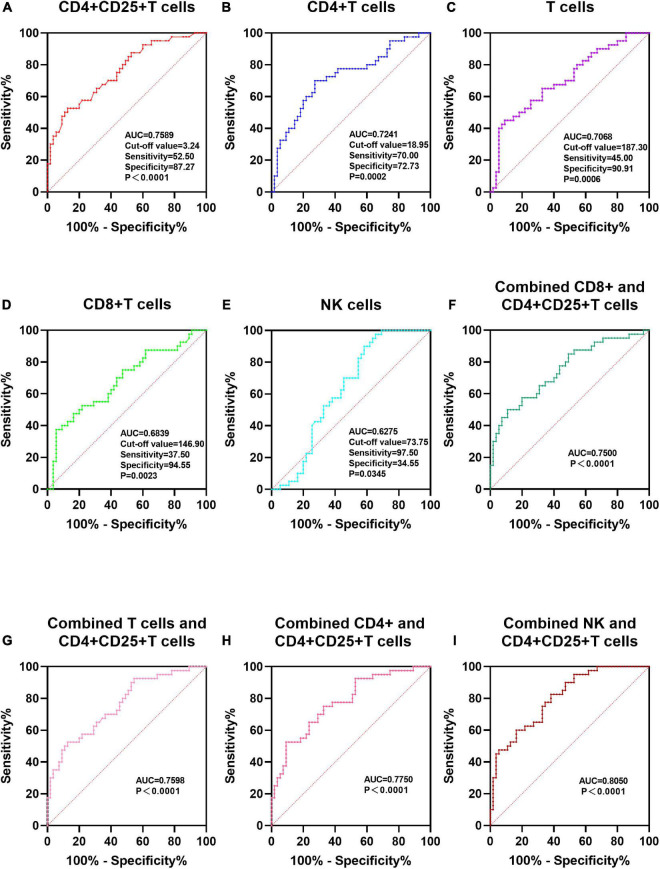
ROC curves of 5 lymphocyte subsets on day + 14 post transplantation predict aGVHD. For each of the five biomarkers, a cut-off value according to aGVHD development was calculated. NK cells, Natural killer cells; **(A–E)** ROC curves of T cells, NK cells, CD4 + T cells, CD4 + CD25 + T cells and CD8 + T cells; **(F–I)** ROC of a 2-biomarker panel. ROC curves of combined CD4 + CD25 + T cells and CD8 + T cells, CD4 + CD25 + T cells and T cells, CD4 + CD25 + T cells and CD4 + T cells, CD4 + CD25 + T cells, and NK cells.

ROC curves and associated area under the curve (AUC) analyses confirmed the association between T cells, CD4 + T cells, CD8 + T cells, activated CD4 + T cells, and NK cells on day + 14 post-transplantation (Table 7 and Figure 4). AUC of NK cells, CD8 + T cells, T cells, CD4 + T cells, and CD4 + CD25 + T cells were 0.6275, 0.6839, 0.7068, 0.7241, and 0.7589, and cut-off values were 73.75 (97.50, 34.55), 146.90 (37.50, 94.55), 187.30 (45.00, 90.91), 18.95 (70.00, 72.73), and 3.24 (52.50, 87.27), respectively (Table 7 and Figures 4A–E). ROC curves analyses of combined CD4 + CD25 + T cells and CD8 + T cells, CD4 + CD25 + T cells and T cells, CD4 + CD25 + T cells and CD4 + T cells, CD4 + CD25 + T cells, and NK cells, were carried out to predict aGVHD and the AUC of the combinations, respectively, were 0.7500, 0.7598, 0.7750, and 0.8050 (*P*<0.0001) (Table 7 and Figures 4F-I).

**TABLE 7:** Analyses of ROC curves of 5 lymphocyte subsets on day + 14 post transplantation predicted aGVHD.

	Cut-off value	Sensitivity	Specificity	AUC	*P*
		(%)	(%)		
NK cells (A)	<73.75/μl	97.50	34.55	0.6275	0.0345
CD8 + T cells (B)	>146.90/μl	37.50	94.55	0.6839	0.0023
T cells (C)	>187.30/μl	45.00	90.91	0.7068	0.0006
CD4 + T cells (D)	>18.95/μl	70.00	72.73	0.7241	0.0002
CD4 + CD25 + T cells (E)	>3.24/μl	52.50	87.27	0.7589	<0.0001
Combined B and E		50.00	89.09	0.7500	<0.0001
Combined C and E		52.50	87.27	0.7598	<0.0001
Combined D and E		52.50	90.91	0.7750	<0.0001
Combined A and E		82.50	61.82	0.8050	<0.0001

A, NK cells; B, CD8 + T cells; C, T cells; D, CD4 + T cells; E, CD4 + CD25 + T cells; AUC, Associated area under the curve.

## Discussion

The pathophysiology of aGVHD is a complex process that can be divided into three phases. Initially, pathogen-associated molecular patterns (PAMPs) and damage-associated molecular patterns (DAMPs) are released from tissues, in response to the chemotherapy or radiotherapy regimen. DAMPs and PAMPs are recognized by innate immune receptors. This interaction leads to the release of pro-inflammatory cytokines (“cytokine storm”), such as TNF-α, IL-1β, and IL-6, which in turn, activate host antigen-presenting cells (28, 29). In the second phase, the interaction of donor T cells with activated APCs expressing MHC and minor host histocompatibility antigens leads to the activation and expansion of T cells (30). In the third phase, named the effector phase, activated donor T cells and monocytes migrate to aGVHD target organs and stimulate the recruitment of other effector cells, such as cytotoxic T cells and natural killer (NK) cells. These effector cells cause damage through direct cytotoxicity or by releasing large amounts of pro-inflammatory cytokines and chemokines, which aggravate aGVHD (30–32). Animal models show that the transition from aGVHD initiation to the aGVHD effector phase can last up to 2 weeks before the first clinical signs of aGVHD appear (33).

In this study, samples were detected on day + 14 post-transplantation in an attempt to predict the occurrence of aGVHD early. There was no difference in the level of each cell subset before transplantation between non-aGVHD and aGVHD groups. Note the effect of pre-transplantation subpopulation levels on post-transplantation levels was excluded. Because T cells proliferate in the second phase of aGVHD, the T cell levels in the aGVHD group were significantly higher than that in the non-aGVHD group (140.36 vs. 69.73). As far as we know, an infection can lead to changes in the level of T lymphocyte subsets and an increase in T cell levels. In this study, there was no significant difference in the constituent ratio of infection that occurred during peri-transplantation between the aGVHD group and the non-aGVHD group, which eliminated the influence of infection factors on the results of the study. Both helper T cells and cytotoxic T cells proliferate in aGVHD. Therefore, the levels of CD4 + and CD8 + T cells were also increased in the aGVHD group. Some reports show that adult and pediatric patients with aGVHD have higher ratios of CD4 + /CD8 + T cells (27, 34, 35). Here, the ratio of CD4 + /CD8 + T cells was higher in the aGVHD group than in the non-aGVHD group. However, the difference was not statistically significant (*P* = 0.105), which was inferred to be related to the insufficient sample size. B cell levels did not differ between the two groups, suggesting that the role of B cells in the pathogenesis of acute graft- vs.-host disease is not key. In previous studies, it was shown that a delayed reconstitution of the NK cells was observed in patients with aGVHD and an inverse relationship between NK-cell levels and aGVHD onset was found (36). Additionally, NK cell levels after allogeneic HSCT have been shown to be predictive of aGVHD (37). Similar results were observed for NK cell levels in this retrospective analysis.

The activation of T cells plays a key role in the development of aGVHD. Cytokine secretion is the main manifestation of T cell activation, and an important cytokine produced by naive T cells is interleukin 2 (IL-2). IL-2 receptor α-chain, also known as CD25, is one of the biomarkers of T cell activation. When activated, T cells express CD25 (38). Results showed that serum cytokine CD25 increased significantly before the onset of aGVHD. But whether or not the level of CD25 + T cells in peripheral blood increased before the onset of aGVHD is unknown. In previous studies, CD25 + T cells were not found to be increased in mouse models (33). However, our data showed that the absolute concentrations of CD4 + CD25 + T cells in the aGVHD group and non-aGVHD were 4.08 (0.91, 11.12) and 0.52 (0.17, 2.20), respectively. The level of CD4 + CD25 + T cells in the aGVHD group was significantly higher on day + 14 post-transplantation, indicating that activated T cells increased before the onset of aGVHD.

The level of CD8 + T cells in the grade I subgroup was significantly higher than in grade II-IV [128.21 (61.11, 258.91) vs. 60.81 (21.59, 176.38), *P* = 0.05] on day + 14 post-transplantation. Lower CD8 + T cell concentrations could likely be a biomarker for severe aGVHD after HSCT. However, a larger sample size is necessary to make a more definitive conclusion.

The predictive value of a single biomarker is often limited. To increase reliability, a composite panel consisting of several biomarkers has a more promising predictive value. We found that a panel of two biomarkers, CD4 + CD25 + T cell levels and NK cell levels, was predictive of aGVHD occurrence with high significance. These markers are relatively easy to determine and should be available in most clinical laboratories. Although the 2-biomarker panel showed high predictive potential for aGVHD, it could not be used to predict the severity of aGVHD.

In summary, our data supported the hypothesis that lymphocyte subsets of peripheral blood are predictors of aGVHD after HSCT in children with TDTβ. We demonstrated that the level of activated CD4 + T cells is elevated in patients with aGVHD and can be used as a biomarker for early prediction of aGVHD. Additionally, the combination of NK cells and activated CD4 + T cells appears to have greater predictive power. Further studies with larger sample sizes are required for validation of using these candidate biomarkers in routine clinical practice.

## Data availability statement

The original contributions presented in this study are included in the article/supplementary material, further inquiries can be directed to the corresponding author.

## Ethics statement

The studies involving human participants were reviewed and approved by the Medical Ethics Committee of First Affiliated Hospital of Guangxi Medical University. Written informed consent to participate in this study was provided by the participants’ legal guardian/next of kin.

## Author contributions

JL contributed to the conception and design of the study. KH organized the database, performed the statistical analysis, and wrote the first draft and sections of the manuscript. Both authors contributed to manuscript revision, read, and approved the submitted version.
